# Monitoring of the oncological process for lung cancer in Spain: an expert consensus report

**DOI:** 10.1007/s12094-025-03883-4

**Published:** 2025-04-22

**Authors:** Javier de Castro, Patricia Alonso-Fernández, José Javier Castrodeza, Ángel Gayete, Florentino Hernando, José Martínez-Olmos, Bartomeu Massuti, Luis Paz-Ares, Antoni Sisó-Almirall, David Vicente, Laureano Molins

**Affiliations:** 1 Department of Medical Oncology, Hospital Universitario La Paz, IdiPaz, Madrid, Spain; 2 Department of Admissions, Hospital Clínico San Carlos, Madrid, Spain; 3 Department of Preventive Medicine, Hospital Clínico Universitario de Valladolid, Castilla y León, Valladolid, Spain; 4 Departament of Diagnostic Radiology, Hospital del Mar de Barcelona, Cataluña, Barcelona, Spain; 5 Department of Thoracic Surgery, Hospital Clínico San Carlos, Madrid, Spain; 6 Escuela Andaluza de Salud Pública (EASP), Andalucía, Spain; 7 Department of Medical Oncology, Hospital General Universitario de Alicante, Valencia, Spain; 8 Department of Medical Oncology, Hospital Universitario 12 Octubre, Madrid, Spain; 9 Consorci d’Atenció Primària de Salut Barcelona Esquerra (CAPSBE), Cataluña, Barcelona, Spain; 10 Department of Medical Oncology, Hospital Universitario Virgen Macarena, Sevilla, Andalucía, Spain; 11 Department of Thoracic Surgery, Hospital Clínic, Barcelona University, Cataluña, Barcelona, Spain

**Keywords:** Monitoring, Oncological process, Lung cancer, Interoperability, Quality, Indicators

## Abstract

**Introduction:**

Continuous monitoring of the oncological process is essential for identifying inefficiencies and areas of improvement, enabling better resource allocation in the care of lung cancer patients.

**Objective:**

The objective is to define key indicators and identify critical variables for monitoring lung cancer care, aiming to improve early detection, reduce delays in diagnosis and treatment, and enhance biomarker research, ensuring timely and effective treatments for all patients.

**Methods:**

A multidisciplinary expert group conducted a consensus process based on a review of national guidelines and initiatives related to lung cancer care. The experts defined relevant indicators and identified variables for monitoring overall care, addressing delays, and improving biomarker research. The feasibility of incorporating these indicators into existing information systems was also assessed.

**Results:**

The proposed indicators provide a structured approach for assessing lung cancer care and outcomes. Their inclusion in healthcare information systems would improve the monitoring and evaluation of care quality and patient outcomes. Additionally, these indicators would also promote interoperability and continuous patient care across different centers and regions, allowing informed decision-making in the improvement of healthcare processes by those responsible for healthcare management.

**Conclusions:**

The adoption of standardized indicators for lung cancer care monitoring can drive continuous improvement in healthcare processes. Implementing these indicators in information systems will enable better resource allocation, timely and effective treatment, and enhanced coordination among healthcare providers, ultimately improving patient outcomes.

**Supplementary Information:**

The online version contains supplementary material available at 10.1007/s12094-025-03883-4.

## Introduction

Lung cancer represents a major public health challenge in Spain, being the leading cause of cancer-related deaths, with a 5-year survival rate of 12.7% in men and 17.6% in women, reported between 2008 and 2013[1]. Its high mortality rate is mainly due to late diagnosis, which reduces the success rate of therapies and chances of survival. Research suggests that lung cancer screening would allow up to 80% of tumors to be diagnosed at early stages, reducing lung cancer mortality by 18%–39%[2–7]. The latest recommendation stated by the United States Preventative Service Task Force (USPSTF) is to perform annual lung cancer screenings with low-dose computed tomography (LDCT) in adults aged 50–80 years, who have a 20-pack-year smoking history and currently smoke or have quit within the past 15 years [8]. Consequently, implementing early detection strategies is essential in the fight against this disease.

Likewise, an effective healthcare response requires rapid and coordinated action to ensure equitable access to quality healthcare. This includes providing an accurate diagnosis and an appropriate treatment as soon as possible. In this regard, advanced molecular diagnosis is crucial in determining tumor malignancy, identifying potential therapeutic targets, enabling personalized treatment options, and monitoring the tumor's response [9].

Continuous monitoring of the oncological process can facilitate the identification of inefficiencies and areas that require improvement, allowing optimal allocation of resources for lung cancer patient care. To achieve this, standardized and protocolized data collection across the National Health System's Digital Health Records is necessary to ensure interoperability and easy access to relevant clinical information, guaranteeing continuity of care even when patients move between centers or Autonomous Communities [10–12]. Furthermore, this will promote coordination among healthcare professionals, encouraging a multidisciplinary approach and collaborative clinical decision-making tailored to each patient [13, 14].

Lastly, this information is essential to support informed decision-making between those responsible for care management processes and health policy, ensuring quality and equity at various levels, from health services and centers to the broader health administration.

Hence, the objective of this project is to define key indicators and standard values and identify variables that are essential to monitor the oncological process, specifically for lung cancer.

## Methods

### Advisory committee and working groups

The Advisory Committee of the Lung Ambition Alliance in Spain, an independent group comprising 24 professionals (including patient representatives and healthcare professionals from different specialties such as pneumology, oncology, thoracic surgery, radiology, pathological anatomy, family medicine, and public health, among others), has been consistently focused on improving the quality of lung cancer care and promoting early detection. Since 2020, the Committee has developed two key documents with recommendations to enhance early detection and ensure equitable access to innovative medicine for precise diagnosis at the earliest stage.

In alignment with efforts to strengthen the Spanish National Healthcare System, the Committee defined key indicators and standard values, identifying essential variables to effectively monitor the oncological process, particularly in lung cancer. To carry out this report, the Advisory Committee was separated into three working groups. Each group worked on defining indicators and variables that allow monitoring of the oncological process of lung cancer. The first group worked on the general monitoring of the oncological process, providing a comprehensive overview of the oncological process. The second group focused on the monitoring of diagnosis and treatment access times within the oncological process of lung cancer. The third group worked on monitoring the implementation of diagnosis tests and the study of lung cancer biomarkers. All members of the three groups received an information dossier outlining the project's objectives, methodology, and their potential responsibilities during its development.

Participation in the project was voluntary and non-confidential; each expert confirmed their involvement by signing a collaboration agreement. All participants were financially compensated based on the time they dedicated to the project.

To develop the report, the indicators and recommendations were first identified through a comprehensive review of relevant literature and analysis. This process was subsequently supported by a series of group meetings and Committee workshops, which employed a structured methodology for brainstorming and decision-making.

### Bibliographic review and analysis

To ensure a comprehensive understanding of the current advancements and challenges in monitoring the oncological process of lung cancer, a detailed literature search was conducted. For the initial proposal of indicators and/or variables, the documents analyzed were [10, 13–19]:

- Consensus and recommendation guidelines concerning the collection of clinical information on the cancer process, particularly lung cancer.
- National legislative documents related to the standardized collection of clinical information in the National Health System.
- Healthcare strategies and policies addressing oncological management.

### Pre-work: identification of indicators and variables

Each group worked collaboratively, through virtual meetings, to define and establish a set of indicators and variables. These indicators were then classified into two categories, based on the experts’ criteria: necessary or desirable. Indicators classified as necessary were those considered essential for the correct monitoring of the oncological process and that are supported by published scientific evidence. Indicators categorized as desirable, although might be more difficult to measure given the current structure of the healthcare information system, are positively valued and should be actively considered for inclusion. Finally, whenever possible, standard values derived from relevant literature were incorporated [16, 19] and, in some instances, adjusted based on the experts’ insights and real-world practice considerations.

### Workshop 1: review and discussion of the identified indicators and variables

In the first workshop, the indicators and variables identified by each working group were presented to all the members of the Advisory Committee of the Lung Ambition Alliance in Spain. The objective of this meeting was for the experts to discuss and evaluate the first proposal of indicators and variables considering different perspectives and points of view from different specialties and fields. The Committee refined each of the elements identified based on their experience and clinical practice.

### Working group meetings

After discussion, the working groups met virtually for the validation of the indicators and variables of each subgroup, making small modifications in the wording and proposal of standard values of the indicators.

### Workshop 2: consensus on indicators and variables for the oncological process of lung cancer

A representative of each group presented the indicators and variables for final validation. Following an iterative process of feedback and refinement, the Advisory Committee of the Lung Ambition Alliance in Spain engaged in discussions and deliberations, where group representatives, acting as moderators, facilitated the exchange of perspectives and provided targeted recommendations. In line with the Recommendation Generation-Based Consensus Model [20], the Committee iteratively refined the indicators and variables, progressively adjusting their preferences based on expert input and feedback until they reached a shared decision.

It should be highlighted that after the consensus meeting, the resulting report was sent to the experts for review. Several rounds of online revisions were conducted, including minor editorial changes and expert comments.

## Results

Below are a series of indicators and standard values, associated with specific variables (Supplementary Tables 1–14), that should be collected and studied within the healthcare system to monitor the oncological process of lung cancer.

### General monitoring of the lung cancer process

Four indicators for epidemiologic surveillance of lung cancer in Spain were proposed (Table 1).

**Table 1:** Indicators for the epidemiologic surveillance of lung cancer

No	Indicator	Necessary/desirable
1	Proportion (%) of patients diagnosed with lung cancer in each stage: I, II, III, or IV	Necessary
2	Proportion (%) of patients diagnosed with lung cancer free of disease by stage:	Necessary
	· Stage I: free of disease	
	· Stage II: free of disease	
	· Stage III: free of disease or progression	
3	Proportion (%) of patients diagnosed with recurrent lung cancer or local recurrence at 5 years	Necessary
4	Proportion (%) of patients diagnosed with recurrent lung cancer or systemic recurrence at 5 years	Necessary

### Monitoring the integration, coordination, and quality of healthcare in the oncological process

To monitor the integration and coordination of the healthcare providers, 17 indicators were proposed (Table 2), including 14 to assess patient access to multidisciplinary teams and psychosocial services.

**Table 2:** Indicators for monitoring the quality and coordination of the healthcare system

No	Indicator	Necessary/desirable	Standard Value [source]
Indicators related to the quality of healthcare			
5	Proportion (%) of molecular diagnoses performed at the center or hospital	Necessary	(−)
6	Proportion (%) of molecular diagnoses outsourced for analysis in another center or hospital	Necessary	(−)
7	Proportion (%) of patients with lung cancer included in a clinical trial or research project	Desirable	(−)
8	Proportion (%) of lung cancer cases that are evaluated in MDT/multidisciplinary thoracic tumor committee, recording their decisions	Necessary	100%^a^ [L]
8.1	Proportion (%) of lung cancer cases that are evaluated in MDTs, recording their decisions	Necessary	< 10% [E]
8.2	Proportion (%) of lung cancer cases that are evaluated in multidisciplinary thoracic tumor committees, recording their decisions *If the hospital or center has a multidisciplinary tumor committee specialized in lung cancer	Necessary	> 90% [E]
9	Proportion (%) of patients with lung cancer, smokers, who have access to smoking cessation programs at the center or hospital	Necessary	> 95% [E]
10	Proportion (%) of patients with lung cancer, smokers, who participate in smoking cessation programs available at the center or hospital	Necessary	50% [E]
11	Proportion (%) of patients with lung cancer, smokers who, after accessing smoking cessation programs, manage to maintain smoking cessation for more than 12 months	Necessary	30–35% [E]
12	Proportion (%) of patients who, after confirmation of the lung cancer diagnosis, have access to psychosocial support resources	Necessary	(−)
13	Proportion (%) of patients who, after confirmation of the diagnosis of lung cancer, have access to respiratory rehabilitation programs or services	Necessary	(−)
14	Time of loss of work–life	Necessary	(−)
15	Proportion (%) of patients with lung cancer with a recognized degree of disability	Necessary	(−)
16	Proportion (%) of patients with lung cancer who have completed a quality-of-life questionnaire	Desirable	(−)
Indicators related to integration and care coordination			
17	Proportion (%) of patients with lung cancer who have a report(s) that includes the care procedures performed during the medical visit or consultation accessible at the different levels of healthcare	Necessary	> 90% [E]
18	Time elapsed for the report(s) to be available in primary care	Necessary	0 min [E]
19	Time elapsed for the report(s) to be found in the social services centers (dependent on the primary care centers)	Necessary	0 min [E]

(-)Standard value not defined. Source codification: *E* standard value recommended by the experts, *L* standard value derived from relevant literature

^a^Standard value adapted from *quality indicators and excellence requirements for a multidisciplinary lung cancer tumor board by the Spanish Lung Cancer Group*^16^

### Monitoring access times to cancer diagnosis and treatment

**Fig. 1:**
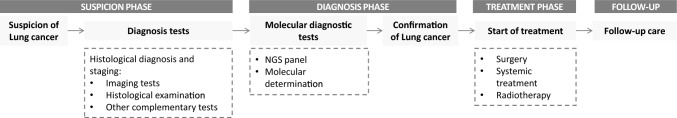
Stages of the oncological process of lung cancer

To monitor the access times to cancer diagnosis and treatment, the experts advocate the inclusion of 38 indicators (Table 3) based on the three phases of the oncological process (Fig. 1). Following the standards defined in the National Cancer Strategy of the Ministry of Health [19], and emphasizing early diagnosis due to late-stage detection, 31 indicators focus on diagnostic access, while 4 address molecular diagnosis and 2 treatment access times.

**Table 3:** Indicators related to access times to diagnosis and treatment

No	Indicator	Necessary /desirable	Standard value
Indicators related to the suspicion phase			
20	Time elapsed from the appearance of the first symptoms to the first medical visit	Necessary	(−)
21	Time elapsed from the first medical visit to the date of consultation with the referred specialist Referred	Necessary	7 days^a^ [L]
22	Time elapsed from the prescription of complementary diagnostic tests until their performance	Necessary	7—20 days [E]
23	Time elapsed from the performance of complementary tests for diagnosis until the issuance of the results report	Necessary	(−)
24	Time elapsed from the issuance of the results report of the complementary tests until the date of the consultation where the patient is informed	Necessary	(−)
25	Time elapsed from the prescription of a chest X-ray until its performance	Necessary	(−)
26	Time elapsed from the performance of a chest X-ray until the issuance of the results report	Necessary	(−)
27	Time elapsed from the issuance of the results report of the chest X-ray until the date of the consultation where the patient is informed	Necessary	(−)
28	Time elapsed from the prescription of a CT scan until its performance	Necessary	7 days [E]
29	Time elapsed from performing a CT scan until the issuance of the results report	Necessary	(−)
30	Time elapsed from the issuance of the CT results report until the date of the consultation where the patient is informed	Necessary	(−)
31	Time elapsed from the prescription of a PET–CT until its performance	Necessary	15 days [E]
32	Time elapsed from the performance of a PET–CT until the issuance of the results report	Necessary	(−)
33	Time elapsed from the issuance of the PET–CT results until the date of the consultation where the patient is informed	Necessary	(−)
34	Time elapsed from the prescription of a bronchoscopy until its performance	Necessary	7 days [E]
35	Time elapsed from performing a bronchoscopy until the issuance of the results report	Necessary	7 days [E]
36	Time elapsed from the issuance of the bronchoscopy results report until the date of the consultation where the patient is informed	Necessary	(−)
37	Time elapsed from the prescription of a mediastinoscopy until its performance	Necessary	20 days [E]
38	Time elapsed from the performance of a mediastinoscopy until the issuance of the results report	Necessary	7 days [E]
39	Time elapsed from the issuance of the mediastinoscopy results report until the date of the consultation where the patient is informed	Necessary	(−)
40	Time elapsed from the prescription of an EBUS until its performance	Necessary	15 days [E]
41	Time elapsed from the performance of an EBUS until the issuance of the results report	Necessary	7 days [E]
42	Time elapsed from the issuance of the EBUS results report until the date of the consultation where the patient is informed	Necessary	(−)
43	Time elapsed from the prescription of an EUS until its performance	Necessary	15 days [E]
44	Time elapsed since performing an EUS until the issuance of the results report	Necessary	7 days [E]
45	Time elapsed from the issue of the EUS results report until the date of the consultation where the patient is informed	Necessary	(−)
46	Time elapsed from the prescription of a brain MRI until its performance	Desirable	7 days [E]
47	Time elapsed from performing a brain MRI until the issuance of the results report	Desirable	(−)
48	Time elapsed from the issuance of the brain MRI results report until the date of the consultation where the patient is informed	Desirable	(−)
49	Time elapsed from the prescription of surgery for a patient suspected of lung cancer until its performance	Necessary	21 days^a^ [L]
50	Time elapsed from the performance of the surgery until the issuance of the pathological report	Necessary	7 days [E]
51	Time elapsed from the issuance of the pathological report until the date of the consultation where the patient is informed	Necessary	(−)
Indicators related to the diagnosis phase			
52	Time elapsed from suspicion of lung cancer to performance of a molecular diagnosis (NGS panel or molecular determination)	Necessary	14 days [E]
53	Time elapsed from the performance of a molecular diagnosis (NGS panel or molecular determination) to the issuance of the molecular report	Necessary	(−)
54	Time elapsed from the issuance of the pathological report to the issuance of the molecular report	Necessary	(−)
55	Time elapsed from suspicion of lung cancer to confirmation of diagnosis	Necessary	15—28 days^a^ [L]
Indicators related to the treatment phase			
56	Time from confirmation of diagnosis to initiation of treatment *Surgery, radiotherapy, or systemic treatment (chemotherapy, targeted therapy, or others)*	Necessary	S and RT: 21 days [E], ST:7 days^a^ [L]
57	Time elapsed from when the treatment plan is defined until the patient and their family are informed	Necessary	5 days [E]

(−)Standard value not defined. Source codification: *E* standard value recommended by the experts, *L* standard value derived from relevant literature

*X-ray* conventional image, *CT* computed tomography, *PET–CT* positron emission tomography and computed tomography, *EBUS* echo-bronchoscopy (endobronchial ultrasound), *EUS* endoscopic ultrasound (endoscopic ultrasound), *MRI* magnetic resonance imaging, *NGS* next-generation sequencing, surgery, *RT* radiotherapy, *ST* systematic treatment

^a^Standard value adapted from the National Cancer Strategy of the National Healthcare System^19^

### Monitoring the implementation of biomarkers for lung cancer

Molecular diagnosis is crucial for determining the best therapeutic strategy for each patient. The Advisory Committee identified 14 indicators (Table 4): 10 to monitor biomarker performance by cancer type and 4 to assess the use of various molecular analysis techniques, based on national consensus to enhance biomarker use in clinical practice.

**Table 4:** Indicators related to monitoring the implementation of biomarker testing for lung cancer

No	Indicator	Necessary /desirable	Standard value
Indicators related to molecular diagnosis			
58	Proportion (%) of patients with NSCLC, non-squamous or squamous subtype, non-smokers and/or younger than 50 years, advanced de novo with an NGS panel that includes: EGFR, ALK, ROS1, RET, NTRK, MET, KRAS, and/or BRAF	Necessary	60% [E]
59	Proportion (%) of patients with NSCLC, non-squamous or squamous subtype, non-smokers and/or under 50 years, advanced de novo with a molecular determination that includes: EGFR, ALK, ROS1, RET, NTRK, MET, KRAS, and/or BRAF	Necessary	100% [E]
60	Proportion (%) of patients with NSCLC, non-squamous or squamous subtype, non-smokers and/or under 50 years, in relapse after locoregional disease, with a molecular determination that includes: EGFR, ALK, ROS1, RET, NTRK, MET, KRAS, and/or BRAF	Necessary	100% [E]
61	Proportion (%) of patients with NSCLC squamous subtype advanced de novo*,* with a molecular determination that includes: NTRK and/or PD-L1	Necessary	100% [E]
62	Proportion (%) of patients with advanced NSCLC treated with TKIs in relapse, with rebiopsy and an NGS panel that includes: EGFR, ALK, ROS1, RET, NTRK, MET, KRAS, and/or BRAF	Necessary	100% [E]
63	Proportion (%) of NSCLC patients with a PD-L1 determination: · Localized NSCLC after radical surgery and adjuvant chemotherapy · Locally advanced NSCLC after radical chemo-radiotherapy treatment · Advanced or metastatic NSCLC	Necessary	100% [E]
64	Proportion (%) of patients with resectable localized NSCLC who undergo molecular determination of EGFR	Necessary	100% [E]
65	Proportion (%) of patients who have been molecularly diagnosed (NGS panel or molecular determination) by liquid biopsy	Necessary	(−)
66	Proportion (%) of patients with lung cancer in whom the necessary tissue sample is not available to perform an NGS panel or molecular determination	Necessary	(−)
67	Proportion (%) of patients with lung cancer who undergo a rebiopsy to perform an NGS panel or molecular determination	Necessary	(−)
Quality indicators related to the distribution of use of analysis techniques for molecular diagnosis, per biomarker			
68	Distribution of the use of analysis techniques for the molecular diagnosis of EGFR, KRAS, and BRAF (percentage of patients with alterations in EGFR, KRAS, and/or BRAF detected by: real-time PCR or NGS)	Necessary	(−)
69	Distribution of the use of analysis techniques for the molecular diagnosis of ALK, ROS1, NTKR, and RET (percentage of patients with alteration in ALK, ROS1, NTKR, and/or RET detected by: IHC, FISH, real-time PCR, or NGS)	Necessary	(−)
70	Distribution of the use of analysis techniques for the molecular diagnosis of MET (percentage of patients with an alteration in MET detected by: FISH, real-time PCR, or NGS)	Necessary	(−)
71	Distribution of the use of analysis techniques for the molecular diagnosis of HER2neu (percentage of patients with HER2neu mutations detected by: IHC, FISH, or NGS)	Desirable	(−)

(−)Standard value not defined. Source codification: *E* standard value recommended by the experts, *L* standard value derived from relevant literature

*NSCLC* non-small cell lung cancer, *PCR* polymerase chain reaction, *IHC* Immunohistochemistry

## Discussion

Lung cancer represents one of the greatest challenges in public healthcare. In Spain, it has a significant strain on the National Healthcare System due to its social, economic, and financial burden. Moreover, it also impacts the psychological and social well-being of patients and their families.

The complexity of the diagnosis and treatment of lung cancer requires a multidisciplinary team of experts to work together to meet patients’ needs. Thus, it is essential to monitor the oncological process by collecting the patients’ information to ensure the efficiency and quality of planning, organizing, and delivering lung cancer care [25]. By analyzing the data generated within the system, healthcare providers and policymakers can identify inefficiencies and specific areas of improvement, promoting equitable access to personalized innovative medicine, and the definition of public healthcare strategies [25]. Additionally, the availability of the information will encourage patients and patient organizations to participate and collaborate in decision-making processes regarding unmet needs.

The Advisory Committee of the Lung Ambition Alliance in Spain reviewed current initiatives, national guidelines, and consensus guidelines to improve cancer care, especially for lung cancer. Based on this review and the Committee members' knowledge, the experts proposed a list of necessary and desirable indicators that should be analyzed within the healthcare system to monitor the oncological process, ensure the continuity of care of lung cancer patients, and support those responsible for health management and policy. The indicators are categorized into three different sets: general monitoring of the oncological process, access times to diagnosis and treatment, and implementation of lung cancer biomarkers.

The first set of indicators focuses on overseeing the overall oncological process. The collection and accessibility of lung cancer patient data across the different digital health records is key to promote coordination between MDTs or multidisciplinary thoracic tumor committees involved in patient care [16, 26]. For the definition of the indicators, the experts considered the excellence requirements and quality indicators for MDTs recommended by the Spanish Lung Cancer Group [16], as well as other consensus documents on the optimal organization and functioning of these teams.

The integration of MDTs in hospitals and health centers has been shown to improve patient care management, and healthcare outcomes, as well as increase the patient’s quality of life and survival [16, 26]. In addition to medical treatment, lung cancer patients may benefit from other services, such as smoking cessation programs, respiratory rehabilitation programs, or psychosocial support programs, among others. Although the medical and scientific community recognize the importance of multidisciplinary cancer care, there is insufficient institutional support for developing high-quality MDTs in Spain. Health authorities should provide them with necessary resources to support their organizational growth and effective functioning.

To ensure that all healthcare providers involved in the process can easily access patients’ clinical information, it is recommended to generate and upload comprehensive and homogeneous reports to the Digital Health Record after each medical consultation. These reports should include, at least, the following sections: reason and date of consultation, diagnosis and date of diagnosis, treatment performed (if applicable), circumstances of last contact, procedures performed, assessment by the MDT, history of the basic process (anamnesis), and recommendations [13, 14, 27].

The second set of indicators is proposed for analyzing and measuring access times to diagnosis and treatment. Early detection of lung cancer has been shown to reduce the overall mortality between 6.7% and 20% [2–6]. Findings of a 2023 study estimated that lung cancer screening with LDCT could reduce mortality from lung cancer by five deaths per 1,000 people (95% CI: 3 to 8). It concluded that lung cancer screening is effective in reducing specific mortality in comparison with non-screening. In this regard, the necessary number of people to be screened (NNS) to avoid one death from lung cancer would be 166 in 10 years [28]. Hence, rapid detection and treatment of lung cancer is crucial for improving the prognosis and survival of patients [26].

The healthcare system must provide timely and adequate diagnosis and treatment while guaranteeing safety, effectiveness, and quality of health services. In the 2021 update of the National Cancer Strategy [19], the goals and strategic directions were outlined to improve cancer prevention and care across the territory. Among the main objectives described was the need to reduce the time between cancer diagnosis and the start of treatment, establishing optimal times for referrals between levels of care and access times to diagnostics tests.

However, the complexity of lung cancer management and possible referrals between centers and services can result in long waiting times for patients [26]. To correct deviations and guarantee equity in access across the territory, the system should rely on mechanisms that monitor real access times throughout the patient’s journey. In this regard, the experts defined five dates that are key and should be registered to oversee the oncological process. These dates include the onset of symptoms, suspicion of lung cancer, prescription for diagnosis tests, confirmation of lung cancer diagnosis, and start of the treatment plan. Additionally, for each of the oncological process phases, standard times are defined according to the National Cancer Strategy guidelines and the experts’ recommendations, based on the knowledge and professional experience.

Lastly, the third set of indicators is proposed for monitoring the implementation and study of biomarkers that allow the definition of an optimal therapeutic strategy. Molecular diagnosis of lung cancer provides information about tumor malignancy and helps to identify possible therapeutic targets for the application of targeted therapy and monitor the tumor response to treatment [7]. While some biomarker tests are mandatory, there is considerable variability in the evaluation and implementation of molecular characterization depending on the type of biomarker, region, and hospital or institution [7]. Given the recent advances in diagnostic techniques, such as the implementation of NGS, and personalized therapies, the Spanish Society of Pathology and the Spanish Society of Medical Oncology published updated consensus guidelines in 2023 for testing predictive lung cancer biomarkers in late stages of the disease. These guidelines update the previous consensus documents from 2012, 2015, and 2020 and aim to set national standards and key milestones, establishing that all hospitals and regions should aspire to include testing for the specified predictive biomarkers for lung cancer [17]. Thus, the Advisory Committee proposed the indicators in the third set based on the guidelines' recommendations and in alignment with the consensus of the Spanish scientific community.

To tackle this situation, the National Healthcare System recently published a common list of biomarkers for oncological diseases and other diseases of a genetic or genomic nature that should be implemented in all hospitals and centers nationwide [18]. Specifically, in the field of lung cancer, this catalog advocates the inclusion of PD-L1, EGFR, ALK, ROS-1, KRAS, NTKR, RET, and MET [18]. Consequently, since 2024, the study of these biomarkers through NGS has been financed by the National Healthcare System for metastatic lung cancer, thus promoting equity in molecular diagnostics throughout the country. It is worth mentioning that, although the BRAF biomarker is not currently listed in the catalog for the molecular study of lung cancer, it is included in the indicators, given its clinical relevance and the development of new therapies directed at this molecular target. It is worth mentioning that the recent introduction of immunotherapy and certain targeted therapies for early and locally advanced stages of the disease makes the incorporation of some of these biomarkers in the non-metastatic setting essential.

In conclusion, the integration of the proposed indicators into the healthcare information systems would not only enhance the monitoring and evaluation of oncological processes but also advocate interoperability and ensure continuous care. This would facilitate patient mobility across different healthcare centers and regions while promoting collaboration, particularly within the European Union. In addition, it would provide reliable data on the healthcare reality in a territory, allowing informed decision-making for health managers and policymakers. Making this information available throughout the healthcare system would enhance resource optimization and the system’s sustainability, while ensuring equitable access to an early diagnosis and personalized treatment and, ultimately, enhancing survival rates and elevating the quality of life for patients.

While the indicators were identified through a review of clinical practice guidelines and expert consensus, it is important to acknowledge the inherent subjectivity derived from the professionals’ diverse experiences and points of view. Notably, these indicators have not been established based on evidence levels, potentially limiting their application in clinical practice. To address this, and as continuity of this study, a national pilot project should be conducted across various hospitals and centers to assess the compliance and effectiveness of the proposed indicators. The pilot project would allow for further analysis and refinement of the indicators before implementing them into healthcare information systems nationwide.

## Supplementary Information

Below is the link to the electronic supplementary material.

Supplementary file1 (DOCX 260 KB)
