# Supplementary material for: Monitoring of the oncological process for lung cancer in Spain: an expert consensus report
Source: Clin Transl Oncol. 2025 Apr 22;27(10):3867–76. doi: 10.1007/s12094-025-03883-4 (PMC12460426; doi:10.1007/s12094-025-03883-4)
Supplement: Supplementary file 1 — Supplementary file1 (DOCX 260 KB) [file 12094_2025_3883_MOESM1_ESM.docx]

**Supplementary Data**

**Monitoring of the Oncological Process for Lung Cancer in Spain: An Expert Consensus Report**

**General patient information**

| **Table 1. Variables related to general patient information** | | | | |
| --- | --- | --- | --- | --- |
| **Code** | | **Variables** | **Values** | **Reference** |
| **1** |  | **Registry Code** **Corresponding Registry Code in the Spanish Cancer Registry Network* |  | (1) |
| **2** |  | **Province** | Dictionary of provinces of the INE | (1) |
| **3** |  | **Tumor identifier code** **Corresponding in the Spanish Cancer Registry Network* |  | (1) |
| **4** |  | **Patient identification code** **Corresponding in the Spanish Cancer Registry Network* |  | (1) |
| **5** | **5.1** | **Tumor sequence number in the patient** | 00 = Single tumor | (1) |
|  | **5.2** |  | 01 = First tumor | (1) |
|  | **5.3** |  | 02 = Second tumor | (1) |
|  | **5.4** |  | Etc. | (1) |
| **6** |  | **Total number of tumors** | No. | (1) |
| **7** | **7.1** | **Sex** | Man | (1) |
|  | **7.2** |  | Women | (1) |
| **8** |  | **Birthdate** | DDMMYYYY | (1) |
| **9** |  | **Incidence date** **The incidence date in the Spanish Cancer Registry Network is considered as the date of confirmation of the diagnosis* | DDMMYYYY | (1) |
| **10** |  | **Year of registration** **Date of registration in the Spanish Cancer Registry Network* | AAAA | (1) |
| **11** |  | **Age at diagnosis** | years | (1) |
| **12** | **12.1** | **Tobacco (at some point in life)** | Yes | (1) |
|  | **12.2** |  | No | (1) |
| **13** |  | **Tobacco consumption start date** | DDMMYYYY  **If you do not have the exact date, indicate only the year (YYYY)* | (*) |
| **14** |  | **Date of cessation of tobacco consumption** | DDMMYYYY  **If you do not have the exact date, indicate only the year (YYYY)* | (*) |
| **15** |  | **Reason for cessation of tobacco use (if applicable)** | Description | (*) |
| **16** | **16.1** | **Tobacco burden** | Non-smoker | (*) |
|  | **16.2** |  | Occasional smoker (<10 packs/year) | (*) |
|  | **16.3** |  | Severe smoker (>10 packs/year) | (*) |
| **17** | **17.1** | **Availability of access to smoking cessation programs in a center or hospital** | Yes | (*) |
|  | **17.2** |  | No | (*) |
| **18** | **18.1** | **Participate in smoking cessation programs at the center or hospital** | Yes | (*) |
|  | **18.2** |  | No | (*) |
| **19** |  | **Program access date** | DDMMYYYY | (*) |
| **20** | **20.1** | **Alcohol (at some point in life)** | Yes | (*) |
|  | **20.2** |  | No | (*) |
| **21** | **21.1** | **Daily alcohol consumption limits** **10 grams are equivalent to 1 shot of 30 ml (alcohol content 40º), half a glass of wine 125ml (alcohol content 10º) or 1 beer of 250ml (alcohol content 5º)  Limits established according to the recommendations of the Organization World Health* | Low risk (< 20 grams/day in men; < 10 grams/day in women) | (*) |
|  | **21.2** |  | High risk (> 40 grams/day in men; > 20 -25 grams/day in women) | (*) |
| **22** | **22.1** | **Availability of access to psychosocial support resources** | Yes | (*) |
|  | **22.2** |  | No | (*) |
| **23** | **23.1** | **Availability of access to respiratory rehabilitation programs or services** | Yes | (*) |
|  | **23.2** |  | No | (*) |
| **24** |  | **Weight (kg)** | kg | (1) |
| **25** |  | **Size (cm)** | cm | (1) |
| **26** | **26.1** | **Body Mass Index (BMI)** | Normal weight | (1) |
|  | **26.2** |  | Overweight | (1) |
|  | **26.3** |  | Obesity | (1) |
|  | **26.4** |  | Underweight | (*) |
| **27** |  | **Medical leave date** | DDMMYYYY | (*) |
| **28** |  | **Medical discharge date** | DDMMYYYY | (*) |
| **29** |  | **Date of granting permanent disability** **The date of granting permanent disability is considered to be the date of granting a permanent disability pension approved by the provincial directorate of Social Security* | DDMMYYYY | (*) |
| The sources marked with (*) are those provided by the members of the Working Groups. | | | | |

**Care coordination**

| **Table 2. Variables related to care coordination** | | | | |
| --- | --- | --- | --- | --- |
| **Code** | | **Variables** | **Values** | **Reference** |
| **30** | **30.1** | **Availability of accessible reports between the different levels of care** **They must be accessible from the different levels of health care (Hospital Care, Primary Care, outpatient clinics, etc.).  Reference is made to the last report prepared* | Yes | (*) |
|  | **30.2** | **Province** | No | (*) |
| **31** |  | **Date of preparation of the report** **Reference is made to the last report prepared* | DDMMYYYY | (*) |
| **32** |  | **Date of publication of the report in Primary Care** **Reference is made to the last report prepared* | DDMMYYYY | (*) |
| **33** |  | **Date of publication of the report in the Social Services Centers** **Reference is made to the last report prepared* | DDMMYYYY | (*) |
| The sources marked with (*) are those provided by the members of the Working Groups. | | | | |

**Patient quality of life**

| **Table 3. Variables related to the patient's quality of life** | | | | |
| --- | --- | --- | --- | --- |
| **Code** | | **Variables** | **Values** | **Reference** |
| **34** | **34.1** | **Comorbidities** | Myocardial infarction (1 pt .) | (1) |
|  | **34.2** |  | Congestive heart failure (1 pt .) | (1) |
|  | **34.3** |  | Peripheral vascular disease (1 pt .) | (1) |
|  | **34.4** |  | Cerebrovascular disease (1 pt .) | (1) |
|  | **34.5** |  | Dementia (1 pt .) | (1) |
|  | **34.6** |  | Chronic obstructive pulmonary disease (1 pt .) | (1) |
|  | **34.7** |  | Rheumatological or connective tissue disease (1 pt .) | (1) |
|  | **34.8** |  | Peptic ulcer (1 pt .) | (1) |
|  | **34.9** |  | Mild liver disease (1 pt .) | (1) |
|  | **34.10** |  | Diabetes without organ damage (1 pt .) | (1) |
|  | **34.11** |  | Hemiplegia or paraplegia (2 pts .) | (1) |
|  | **34.12** |  | Moderate or severe kidney disease (2 pts .) | (1) |
|  | **34.13** |  | Diabetes with organ damage (2 pts .) | (1) |
|  | **34.14** |  | Any solid malignant tumor without metastasis (2 pts .) | (1) |
|  | **34.15** |  | Lymphoma (2 pts .) | (1) |
|  | **34.16** |  | Leukemia (2 pts .) | (1) |
|  | **34.17** |  | Moderate or severe liver disease | (1) |
|  | **34.18** |  | Metastatic solid tumor | (1) |
|  | **34.19** |  | AIDS/HIV | (1) |
|  | **34.20** |  | Others | (1) |
| **35** | **35.1** | **Recognized degree of disability** **As defined in Royal Decree 1971/1999, chapter 11: Neoplasies* | Null (0%) | (*) |
|  | **35.2** |  | Mild (1 - 24%) | (*) |
|  | **35.3** |  | Moderate (25 - 49%) | (*) |
|  | **35.4** |  | Severe (50 - 70%) | (*) |
|  | **35.5** |  | Very serious (>75%) | (*) |
| **36** | **36.1** | **Carrying out a quality-of-life questionnaire** | Yes | (*) |
|  | **36.2** |  | No | (*) |
| **37** |  | **Questionnaire name** | Description | (*) |
| The sources marked with (*) are those provided by the members of the Working Groups. | | | | |

**Systematic population screening**

| **Table 4. Variables related to screening** | | | | | |
| --- | --- | --- | --- | --- | --- |
| **Code** | | | **Variables** | **Values** | **Reference** |
| **38** | **38.1** | **Diagnosed through systematic population screening** | | Yes | (1) |
|  | **38.2** |  |  | No | (1) |
| The sources marked with (*) are those provided by the members of the Working Groups. | | | | | |

**First clinical consultation**

| ***Table 4. Variables related to the first clinical consultation*** | | | | |
| --- | --- | --- | --- | --- |
| **Code** | | **Variables** | **Values** | **Reference** |
| **39** |  | **Date of first medical consultation** | DDMMYYYY | (*) |
| **40** | **40.1** | **Level of care visited and/or specialty (if applicable)** | Primary and Community Care Professional | (*) |
|  | **40.2** |  | Specialist (Primary and Hospital Care) | (*) |
|  | **40.3** |  | Emergencies (Primary and Hospital Care) | (*) |
| **41** |  | **Date of first symptom appearance** | DDMMYYYY | (*) |
| **42** |  | **Symptom** | Description | (*) |
| **43** | **43.1** | **Complementary tests** **In accordance with what is defined in the test folders in the CMDIC* | Laboratory | (2,3)(*) |
|  | **43.2** |  | Image | (2,3)(*) |
|  | **43.3** |  | Others | (2,3)(*) |
| **44** |  | **Date of prescription of complementary tests** | DDMMYYYY | (*) |
| **45** |  | **Date of completion of complementary tests** | DDMMYYYY | (*) |
| **46** |  | **Date of issuance of the report of results of complementary tests** **The date of issue is considered as the moment in which the report of the results is signed* | DDMMYYYY | (*) |
| **47** |  | **Diagnosis** | Description | (*) |
| **48** | **48.1** | **Definition of diagnosis** | Suspicion | (*) |
|  | **48.2** |  | Definitive | (*) |
| **49** |  | **Date of diagnosis (suspected or definitive)** **Date of the medical consultation in which the results are communicated* | DDMMYYYY | (*) |
| **50** | **50.1** | **Conclusion of diagnosis** | Follow-up | (*) |
|  | **50.2** |  | Derivation | (*) |
| **51** |  | **Date of follow-up visit** **In case of follow-up* | DDMMYYYY | (*) |
| The sources marked with (*) are those provided by the members of the Working Groups. | | | | |

**Diagnosis of the neoplasia**

| ***Table 5. Variables related to the diagnosis of neoplasia*** | | | | |
| --- | --- | --- | --- | --- |
| **Code** | | **Variables** | **Values** | **Reference** |
| **52** |  | **Date of visit with specialist** **In case of referral* | DDMMYYYY | (*) |
| **53** | **53.1** | **Referred specialist  **In case of referral*** | Primary Specialist | (*) |
|  | **53.2** |  | Hospital Specialist | (*) |
|  | **53.3** |  | Hospital emergency | (*) |
| **54** | **54.1** | **Imaging tests** | Conventional chest imaging (x-ray) | (1)(*) |
|  | **54.2** |  | Computed Tomography (CT) | (1)(*) |
|  | **54.3** |  | PET or PET-CT (positron emission tomography/computed tomography) | (1)(*) |
|  | **54.4** |  | Brain magnetic resonance imaging (MRI) | (1)(*) |
|  | **54.5** |  | Others | (1)(*) |
| **55** |  | **Imaging test prescription date** | DDMMYYYY | (*) |
| **56** |  | **Date of imaging tests** | DDMMYYYY | (1)(*) |
| **57** |  | **Issue date of the imaging test results report** **The issue date is considered the moment in which the results report is signed* | DDMMYYYY | (*) |
| **58** | **58.1** | **Endoscopic tests** | Bronchoscopy | (1)(*) |
|  | **58.2** |  | Ecobronchoscopy (EBUS, *Endo Bronchial Ultrasound)* | (1)(*) |
|  | **58.3** |  | Endoscopy (EUS, *Endoscopic Ultrasound)* | (*) |
|  | **58.4** |  | Mediastinoscopy | (1)(*) |
|  | **58.5** |  | Other | (1)(*) |
| **59** |  | **Date of indication for endoscopic tests** | DDMMYYYY | (*) |
| **60** |  | **Date of endoscopic tests** | DDMMYYYY | (1)(*) |
| **61** |  | **Date of issue of the endoscopic tests report** **The date of issue is considered the moment in which the report of the results is signed* | DDMMYYYY | (*) |
| **62** |  | **Other tests or procedures** | Type of test | (*) |
| **63** |  | **Date of other tests or procedures** | DDMMYYYY | (*) |
| **64** |  | **Date of issuance of the report of results of other tests or procedures** **The date of issuance is considered the moment in which the results report is signed* | DDMMYYYY | (*) |
| **65** |  | **Definitive diagnosis** | Description | (*) |
| **66** |  | **Date of definitive diagnosis** **The date of definitive diagnosis is considered the date of the medical consultation in which the patient is informed about the results of the tests and the diagnosis* | DDMMYYYY | (*) |
| The sources marked with (*) are those provided by the members of the Working Groups. | | | | |

**Tumor characteristics**

| ***Table 6. Variables related to tumor characteristics*** | | | | |
| --- | --- | --- | --- | --- |
| **Code** | | **Variables** | **Values** | **Reference** |
| **67** |  | **Location of the primary tumor** | According to ICD-O-3 (4 digits) | (1) |
| **68** |  | **Tumor morphology** | According to ICD-O-3 (4 digits) | (1) |
| **69** |  | **Behavior** | According to ICD-O-3 (4 digits) | (1) |
| **70** |  | **Degree** | According to ICD-O-3 (4 digits) | (1) |
| **71** | **71.1** | **Functional status at diagnosis** | Karnosfsky: score from 0 to 100 | (1) |
|  | **71.2** |  | ECOG/WHO: score from 0 to 5 | (1) |
| **72** |  | **Most valid (base) method of diagnosis** | According to ICD-O-3 (4 digits) | (1) |
| **73** |  | **Affected organ** | Description | (*) |
| **74** | **74.1** | **Laterality** **In case of paired organs* | Left | (1) |
|  | **74.2** |  | Right | (1) |
|  | **74.3** |  | Both | (1) |
| **75** |  | **Region** | Description | (*) |
| **76** | **76.1** | **Metastasis** | Yes: location according to ICD-O-3 (4 digits) | (1) |
|  | **76.2** |  | No | (1) |
| The sources marked with (*) are those provided by the members of the Working Groups. | | | | |

**Staging**

| ***Table 7. Variables related to staging*** | | | | |
| --- | --- | --- | --- | --- |
| **Code** | | **Variables** | **Values** | **Reference** |
| **77** |  | **Staging determination date** | DDMMYYYY | (1)(*) |
| **78** | **78.1** | **Staging classification** | TNM | (*) |
|  | **78.2** |  | FIGO | (*) |
|  | **78.3** |  | Others | (*) |
| **79** | **79.1** | **TNM Stadium** | Description: according to TNM-UICC classification | (1)(*) |
|  | **79.2** |  | Clinical doubt: NOS (Not Otherwise Specified) or NOS (Not Otherwise Specified) Otherwise Specified)  *According to ICD-10 | (*) |
| **80** | **80.1** | **TNM Edition** | 05 yes 5th edition | (1) |
|  | **80.2** |  | 06 yes 6th edition | (1) |
|  | **80.3** |  | 07 yes 7th edition | (1) |
|  | **80.4** |  | 08 yes 8th edition | (1) |
| **81** |  | **cT** | According to TNM-UICC Classification | (1) |
| **82** |  | **cN** | According to TNM-UICC Classification | (1) |
| **83** |  | **cm** | According to TNM-UICC Classification | (1) |
| **84** |  | **pT** | According to TNM-UICC Classification | (1) |
| **85** |  | **pN** | According to TNM-UICC Classification | (1) |
| **86** |  | **p.m** | According to TNM-UICC Classification | (1) |
| **87** |  | **Clinical tumor diameter (mm)** | mm | (1) |
| **88** |  | **Pathological tumor diameter (mm)** | mm | (1) |
| **89** |  | **Total number of lymph nodes examined** | No. | (1) |
| **90** |  | **Total number of metastatic lymph nodes** | No. | (1) |
| **91** |  | **Date of issue of the anatomopathological report** **The date of issue is considered the moment in which the report of the results is signed* | DDMMYYYY | (*) |
| The sources marked with (*) are those provided by the members of the Working Groups. | | | | |

**Molecular diagnosis**

| **Table 7. Variables related to molecular diagnosis** | | | | | | | | |
| --- | --- | --- | --- | --- | --- | --- | --- | --- |
| **Code** | | | **Variables** | | **Values** | | **Reference** | |
| **92** | **92.1** | **Making an NGS panel** | | Yes | | (1) | |  |
|  | **92.2** |  |  | No | | (1) | |  |
| **93** |  | **Reason why an NGS panel is not carried out** **If the "no" box is checked* | | Description | | (*) | |  |
| **94** |  | **NGS panel prescription date** | | DDMMYYYY | | (*) | |  |
| **95** |  | **NGS panel completion date** | | DDMMYYYY | | (1) | |  |
| **96** |  | **Issue date of the NGS panel results report (molecular report)** **The issue date is considered the moment in which the results report is signed* | | DDMMYYYY | | (*) | |  |
| **97** | **97.1** | **Carrying out a molecular determination** | | Yes | | (1) | |  |
|  | **97.2** |  |  | No | | (1) | |  |
| **98** |  | **Reason why a molecular determination is not performed  **If the "no" box is checked*** | | Description | | (*) | |  |
| **99** |  | **Prescription date of the molecular determination** | | DDMMAAA | | (*) | |  |
| **100** |  | **Date of performance of a molecular determination** | | DDMMYYYY | | (1) | |  |
| **101** |  | **Issue date of the molecular determination results report (molecular report)** **The issue date is considered the moment in which the results report is signed* | | DDMMYYYY | | (*) | |  |
| **102** | **102.1** | **Associated with a hereditary syndrome** | | Yes | | (1) | |  |
|  | **102.2** |  |  | No | | (1) | |  |
| **103** | **103.1** | **Location of NGS panel or molecular determination** | | Center or hospital | | (*) | |  |
|  | **103.2** |  |  | Referral to another center or hospital | | (*) | |  |
| **104** |  | **Sample collection date** | | DDMMYYYY | | (4) | |  |
| **105** |  | **Sample number** | | No. | | (4) | |  |
| **106** | **106.1** | **Sample obtaining technique** | | Biopsy | | (*) | |  |
|  | **106.2** |  |  | Liquid biopsy | | (4) | |  |
|  | **106.3** |  |  | Core needle biopsy (CNB) | | (*) | |  |
|  | **106.4** |  |  | Cytology | | (4) | |  |
| **107** | **107.1** | **Sample origin** | | Tissue | | (*) | |  |
|  | **107.2** |  |  | Blood | | (*) | |  |
| **108** | **108.1** | **Type of sample** | | Description | | (4)(*) | |  |
|  | **108.2** |  |  | LOINC Nomenclature*: Biochemistry, Hematology, Immunology, Genetics and Microbiology  **LOINC (Logical Observation Identifiers Names and Codes) or translated into Spanish, set of identifiers, names and codes* | | (4)(*) | |  |
|  | **108.3** |  |  | Nomenclature SNOMED -CT*: Anatomy Pathological  **Systematized Nomenclature of Medicine – Clinical Terms* | | (4)(*) | |  |
| **109** | **109.1** | **Rebiopsy** | | Yes | | (*) | |  |
|  | **109.2** |  |  | No | | (*) | |  |
| **110** | **110.1** | **Determination** | | general biochemistry | | (4)(*) | |  |
|  | **110.2** |  |  | Systematic urine | | (4)(*) | |  |
|  | **110.3** |  |  | Hormones | | (4)(*) | |  |
|  | **110.4** |  |  | Tumor markers | | (4)(*) | |  |
|  | **110.5** |  |  | Drug and toxic level | | (4)(*) | |  |
|  | **110.6** |  |  | Hematology blood gas | | (4)(*) | |  |
|  | **110.7** |  |  | Hemostasis (Coagulation) | | (4)(*) | |  |
|  | **110.8** |  |  | Hemotherapy | | (4)(*) | |  |
|  | **110.9** |  |  | Hematology-Coagulation: special tests | | (4)(*) | |  |
|  | **110.10** |  |  | Immunology- Allergy | | (4)(*) | |  |
|  | **110.11** |  |  | Genetic Microbiology | | (4)(*) | |  |
|  | **110.12** |  |  | Pathological Anatomy - Biopsies | | (4)(*) | |  |
|  | **110.13** |  |  | Pathological Anatomy - Cytology | | (4)(*) | |  |
| **111** | **111.1** | **Sample analysis technique** | | Biopsies and surgical pieces | | (4)(*) | |  |
|  | **111.2** |  |  | Immunohistochemistry (IHC) | | (4)(*) | |  |
|  | **111.3** |  |  | Molecular Pathological Anatomy | | (4)(*) | |  |
|  | **111.4** |  |  | Electron microscopy | | (4)(*) | |  |
|  | **111.5** |  |  | Flow cytometry | | (4)(*) | |  |
|  | **111.6** |  |  | Cytologies and Biopsy with fine needle puncture (FNAC) | | (4)(*) | |  |
|  | **111.7** |  |  | Immunocytochemistry | | (4)(*) | |  |
|  | **111.8** |  |  | Flow colometry | | (4)(*) | |  |
|  | **111.9** |  |  | Cytogenetics | | (4)(*) | |  |
|  | **111.10** |  |  | FISH | | (5)(*) | |  |
|  | **111.11** |  |  | NGS | | (5)(*) | |  |
|  | **111.12** |  |  | qPCR (real-time PCR) | | (5)(*) | |  |
| **112** | **112.1** | **Molecular marker(s) studied** **All biomarkers that are studied must be indicated* | | PD-L1 | | (1) | |  |
|  | **112.2** |  |  | EGFR | | (1) | |  |
|  | **112.3** |  |  | ALK | | (1) | |  |
|  | **112.4** |  |  | ROS-1 | | (1) | |  |
|  | **112.5** |  |  | KRAS | | (1) | |  |
|  | **112.6** |  |  | BRAF | | (1) | |  |
|  | **112.7** |  |  | NTRK | | (1) | |  |
|  | **112.8** |  |  | RET | | (1) | |  |
|  | **112.9** |  |  | MET | | (1) | |  |
|  | **112.10** |  |  | Others: TMB, STK11, KEAP1, MSI, KRASg12c, HER2neu, etc. | | (1,5)(*) | |  |
|  | **112.11** |  |  | None | | (1) | |  |
| **113** | **113.1** | **Molecular marker(s) present in the sample** **All biomarkers present must be indicated* | | PD-L1 | | (1) | |  |
|  | **113.2** |  |  | EGFR | | (1) | |  |
|  | **113.3** |  |  | ALK | | (1) | |  |
|  | **113.4** |  |  | ROS-1 | | (1) | |  |
|  | **113.5** |  |  | KRAS | | (1) | |  |
|  | **113.6** |  |  | BRAF | | (1) | |  |
|  | **113.7** |  |  | NTRK | | (1) | |  |
|  | **113.8** |  |  | RET | | (1) | |  |
|  | **113.9** |  |  | MET | | (1) | |  |
|  | **113.10** |  |  | Others: TMB, STK11, KEAP1, MSI, KRASg12c, HER2neu, etc. | | (1,5)(*) | |  |
|  | **113.11** |  |  | None | | (1) | |  |
| **114** | **114.1** | **PD-L1 expression level** | | Negative (<1%) | | (1) | |  |
|  | **114.2** |  |  | Positive (1 to 49%) | | (1) | |  |
|  | **114.3** |  |  | Positive (≥50%) | | (1) | |  |
| The sources marked with (*) are those provided by the members of the Working Groups. | | | | | | | |  |

**First treatment**

| **Table 8 . Variables related to the first treatment** | | | | | | | |
| --- | --- | --- | --- | --- | --- | --- | --- |
| **Code** | | | **Variables** | | **Values** | **Reference** | |
| **115** | **115.1** | **Case evaluated and registered by a Multidisciplinary Tumor Committee** | | Yes | | (1) |  |
|  | **115.2** |  |  | No | | (1) |  |
| **116** |  | **Number of times in which the patient has been evaluated and registered by a Multidisciplinary Tumor Committee** | | No. | | (*) |  |
| **117** | **117.1** | **Case evaluated and registered by a Multidisciplinary Thoracic Tumors Committee** | | Yes | | (*) |  |
|  | **117.2** |  |  | No | | (*) |  |
| **118** |  | **Number of times in which the patient has been evaluated and registered by a Multidisciplinary Thoracic Tumors Committee** | | No. | | (*) |  |
| **119** |  | **Date of definition of the treatment plan** **The date of definition of the treatment plan is considered to be the date on which the Multidisciplinary Committee for Tumors/Thoracic Tumors makes a joint decision in relation to the treatment plan* | | DDMMYYYY | | (*) |  |
| **120** |  | **Date of consultation on decision of the Multidisciplinary Committee on Tumors/Thoracic Tumors** **Date of medical consultation in which the patient is informed about the treatment, treatment plan, etc.* | | DDMMYYYY | | (*) |  |
| **121** |  | **Treatment start date** | | DDMMYYYY | | (*) |  |
| **122** | **122.1** | **Surgery** | | Yes | | (1)(*) |  |
|  | **122.2** |  |  | No | | (1)(*) |  |
| **123** |  | **Surgery prescription date** | | DDMMYYYY | | (*) |  |
| **124** | **124.1** | **Type of surgery** | | Robotic surgery | | (1) |  |
|  | **124.2** |  |  | Video assisted surgery | | (*) |  |
|  | **124.3** |  |  | open surgery | | (*) |  |
| **125** | **125.1** | **Resection type** | | Atypical Segmentectomy | | (*) |  |
|  | **125.2** |  |  | Anatomical Segmentectomy | | (*) |  |
|  | **125.3** |  |  | Bilobectomy | | (*) |  |
|  | **125.4** |  |  | Lobectomy | | (*) |  |
|  | **125.5** |  |  | Pneumonectomy | | (*) |  |
| **126** | **126.1** | **Lymphadenectomy** | | Lymphadenectomy not performed | | (*) |  |
|  | **126.2** |  |  | *Sampling* | | (*) |  |
|  | **126.3** |  |  | Systematic *sampling* | | (*) |  |
|  | **126.4** |  |  | Systematic Mediastinal Dissection (SMD) | | (*) |  |
|  | **126.5** |  |  | Specific Lobe Dissection | | (*) |  |
| **127** | **127.1** | **Surgical radicalism** | | R0, no residual tumor | | (1) |  |
|  | **127.2** |  |  | RUn, *Uncertain* | | (*) |  |
|  | **127.3** |  |  | R1, microscopic residual tumor | | (1) |  |
|  | **127.4** |  |  | R2, macroscopic residual tumor | | (1) |  |
|  | **127.5** |  |  | Not resectable | | (1) |  |
| **128** | **128.1** | **Reasons for not surgery** | | Not indicated | | (1) |  |
|  | **128.2** |  |  | Medical contraindication | | (1) |  |
|  | **128.3** |  |  | Patient refusal | | (1) |  |
|  | **128.4** |  |  | Others | | (1) |  |
| **129** | **129.1** | **Chemotherapy** | | Yes | | (1)(*) |  |
|  | **129.2** |  |  | No | | (1)(*) |  |
|  | **129.3** |  |  | Does not apply | | (1)(*) |  |
| **130** | **130.1** | **Type of chemotherapy** | | Platinum-based chemotherapy | | (1) |  |
|  | **130.2** |  |  | Non-platinum chemotherapy | | (1) |  |
|  | **130.3** |  |  | Chemotherapy + immunotherapy | | (1) |  |
|  | **130.4** |  |  | Chemotherapy + antiangiogenic | | (1) |  |
|  | **130.5** |  |  | Chemotherapy + immunotherapy + antiangiogenic | | (1) |  |
|  | **130.6** |  |  | Monotherapy immunotherapy | | (1) |  |
|  | **130.7** |  |  | Immunotherapy doublet | | (1) |  |
| **131** | **131.1** | **Chemotherapy intention** | | Healing | | (*) |  |
|  | **131.2** |  |  | Neoadjuvant | | (1) |  |
|  | **131.3** |  |  | Adjuvant | | (1) |  |
|  | **131.4** |  |  | Perioperative | | (1) |  |
|  | **131.5** |  |  | Radical concomitant with radiotherapy | | (1) |  |
|  | **131.6** |  |  | Sequential radical with radiotherapy | | (1) |  |
|  | **131.7** |  |  | Advanced illness | | (1) |  |
| **132** | **132.1** | **Targeted treatment** | | Yes | | (1) |  |
|  | **132.2** |  |  | No | | (1) |  |
|  | **132.3** |  |  | Does not apply | | (1) |  |
| **133** | **133.1** | **Type of targeted treatment** | | Osimertinib | | (1) |  |
|  | **133.2** |  |  | Gefitinib | | (1) |  |
|  | **133.3** |  |  | Erlotinib | | (1) |  |
|  | **133.4** |  |  | Afatinib | | (1) |  |
|  | **133.5** |  |  | Dacomitinib | | (1) |  |
|  | **133.6** |  |  | Crizotinib | | (1) |  |
|  | **133.7** |  |  | Ceritinib | | (1) |  |
|  | **133.8** |  |  | Alectinib | | (1) |  |
|  | **133.9** |  |  | Brigatinib | | (1) |  |
|  | **133.10** |  |  | Clinical trial | | (1) |  |
|  | **133.11** |  |  | Other | | (1) |  |
| **134** | **134.1** | **Radiotherapy** | | Yes | | (1)(*) |  |
|  | **134.2** |  |  | No | | (1)(*) |  |
|  | **134.3** |  |  | Does not apply | | (1)(*) |  |
| **135** | **135.1** | **Radiotherapy modality (multiple response)** | | 3D | | (*) |  |
|  | **135.2** |  |  | Intensity Modulated Radiotherapy (IMRT)/ Volumetric Intensity Modulated Arctherapy (VMAT) | | (*) |  |
|  | **135.3** |  |  | Stereotactic Body Radiation Therapy (SBRT) | | (*) |  |
| **136** | **136.1** | **Intention of radiotherapy** | | Neoadjuvant | | (*) |  |
|  | **136.2** |  |  | Adjuvant | | (*) |  |
|  | **136.3** |  |  | Exclusive Radical | | (*) |  |
|  | **136.4** |  |  | Radical concomitant with systemic treatment | | (*) |  |
|  | **136.5** |  |  | Sequential radical with systemic treatment | | (1) |  |
|  | **136.6** |  |  | Palliative | | (1) |  |
|  | **136.7** |  |  | Brain prophylaxis | | (1) |  |
|  | **136.8** |  |  | Other | | - |  |
| **137** | **137.1** | **Reasons not to do radiotherapy** | | Medical contraindication | | (1) |  |
|  | **137.2** |  |  | Patient refusal | | (1) |  |
|  | **137.3** |  |  | Not indicated | | (1) |  |
|  | **137.4** |  |  | Other | | (1) |  |
| **138** | **138.1** | **Percutaneous treatments** | | Yes | | (*) |  |
|  | **138.2** |  |  | No | | (*) |  |
| **139** |  | **Type of percutaneous treatment** | | Description | | (*) |  |
| **140** |  | **Other treatments** | | Description | | (*) |  |
| **141** | **141.1** | **Refusal to treatment** | | Yes | | (1) |  |
|  | **141.2** |  |  | No | | (1) |  |
| **142** | **142.1** | **Participation in a clinical trial or research project** | | Yes | | (1) |  |
|  | **142.2** |  |  | No | | (1) |  |
| **143** |  | **End date of first treatment** | | DDMMYYYY | | (1)(*) |  |
| The sources marked with (*) are those provided by the members of the Working Groups. | | | | | | |  |

**Response to first treatment**

| **Table 9 . Variables related to the response to the first treatment** | | | | |
| --- | --- | --- | --- | --- |
| **Code** | | **Variables** | **Values** | **Reference** |
| **144** | **144.1** | **Response type** *According to RECIST (*Response Evaluation) characterization Criteria in Solid Tumors)* | Complete answer | (1)(*) |
|  | **144.2** |  | Partial answer | (1)(*) |
|  | **144.3** |  | Stable disease | (1)(*) |
|  | **144.4** |  | Progressing disease | (1)(*) |
|  | **144.5** |  | Does not apply | (1)(*) |
|  | **144.6** |  | Not evaluable | (1)(*) |
| **145** | **145.1** | **Evolution of the disease** | Disease-free patient | (*) |
|  | **145.2** |  | Progression-free patient | (*) |
|  | **145.3** |  | Stable patient | (*) |
|  | **145.4** |  | Patient in progression | (*) |
|  | **145.5** |  | Refractory or metastatic disease | (*) |
|  | **145.6** |  | Other | (*) |
| The sources marked with (*) are those provided by the members of the Working Groups. | | | | |

**Subsequent treatment(s)**

| ***Table 10 . Variables related to successive treatments*** | | | | |
| --- | --- | --- | --- | --- |
| **Code** | | **Variables** | **Values** | **Reference** |
| **146** | **146.1** | **Recurrence or relapse** | Yes | (1)(*) |
|  | **146.2** |  | No | (1)(*) |
| **147** | **147.1** | **Type of recurrence or relapse** | Local recurrence | (1) |
|  | **147.2** |  | Regional recurrence | (1) |
|  | **147.3** |  | Systemic recurrence (distant metastasis) | (1) |
|  | **147.4** |  | Others | (1) |
| **148** |  | **Confirmation date of recurrence or relapse** | DDMMYYYY | (1)(*) |
| **149** |  | **Treatment start date** | DDMMYYYY | (*) |
| **150** | **150.1** | **Type of treatment** | Surgery: procedure | (*) |
|  | **150.2** |  | Chemotherapy: drug/s | (*) |
|  | **150.3** |  | Immunotherapy: drug/s | (*) |
|  | **150.4** |  | Radiotherapy: procedure | (*) |
|  | **150.5** |  | Percutaneous techniques: procedure | (*) |
|  | **150.6** |  | Others: description | (*) |
| **151** |  | **Treatment end date** | DDMMYYYY | (1) |
| **152** |  | **Response type** | Response type: according to the RECIST characterization (Response *Evaluation Criteria in Solid Tumors)* | (1) |
| **153** | **153.1** | **Evolution of the disease** | Disease-free patient | (*) |
|  | **153.2** |  | Progression-free patient | (*) |
|  | **153.3** |  | Stable patient | (*) |
|  | **153.4** |  | Patient in progression | (*) |
|  | **153.5** |  | Refractory or metastatic disease | (*) |
|  | **153.6** |  | Other | (*) |
| The sources marked with (*) are those provided by the members of the Working Groups. | | | | |

**Response to completion of last treatment**

| **Table 11 . Variables related to the response to the last treatment** | | | | |
| --- | --- | --- | --- | --- |
| **Code** | | **Variables** | **Values** | **Reference** |
| **154** |  | **Last treatment start date** | DDMMYYYY | (1) |
| **155** |  | **End date of last treatment** | DDMMYYYY | (1) |
| **156** |  | **Response type** | Response type: according to the RECIST characterization (Response *Evaluation Criteria in Solid Tumors)* | (1)(*) |
| **157** | **157.1** | **Evolution of the disease** | Disease-free patient | (*) |
|  | **157.2** |  | Progression-free patient | (*) |
|  | **157.3** |  | Stable patient | (*) |
|  | **157.4** |  | Patient in progression | (*) |
|  | **157.5** |  | Refractory or metastatic disease | (*) |
|  | **157.6** |  | Other | (*) |
| The sources marked with (*) are those provided by the members of the Working Groups. | | | | |

**Follow-up**

| **Table 12 . Variables related to patient follow-up** | | | | |
| --- | --- | --- | --- | --- |
| **Code** | | **Variables** | **Values** | **Reference** |
| **158** |  | **Last contact date** | DDMMYYYY | (1)(*) |
| **159** | **159.1** | **Vital status at last known contact** | Alive | (1) |
|  | **159.2** |  | Deceased | (1) |
| The sources marked with (*) are those provided by the members of the Working Groups. | | | | |

**Progression**

| ***Table 13 . Variables related to disease progression*** | | | | |
| --- | --- | --- | --- | --- |
| **Code** | | **Variables** | **Values** | **Reference** |
| **160** |  | **Second cancer** | Description | (1) |
| **161** |  | **Date of diagnosis of second cancer** | DDMMYYYY | (1) |
| **162** | **162.1** | **Diagnostic method** | Imaging tests | (*) |
|  | **162.2** |  | Endoscopy | (*) |
|  | **162.3** |  | Molecular diagnosis | (*) |
|  | **162.4** |  | Other tests or procedures | (*) |
| **163** |  | **Date of diagnostic tests** | DDMMYYYY | (*) |
| **164** |  | **Date of receipt of test results** | DDMMYYYY | (*) |
| **165** | **165.1** | **Diagnostic Test Results** | Imaging tests | (*) |
|  | **165.2** |  | Endoscopy | (*) |
|  | **165.3** |  | Molecular diagnosis | (*) |
|  | **165.4** |  | Other tests or procedures: type of test | (*) |
| **166** |  | **Staging determination date** | DDMMYYYY | (*) |
| **167** | **167.1** | **Classification staging** | TNM | (*) |
|  | **167.2** |  | FIGO | (*) |
|  | **167.3** |  | Others | (*) |
| **168** | **168.1** | **TNM Stadium** | Description: according to TNM-UICC classification | (1)(*) |
|  | **168.2** |  | Clinical doubt: NOS (Not Otherwise Specified) or NOS (*Not Otherwise Specified) Otherwise Specified*)  *According to ICD-10 | (*) |
| **169** | **169.1** | **Carrying out a molecular determination** | Yes | (*) |
|  | **169.2** |  | No | (*) |
| **170** |  | **Number of molecular determinations performed on the patient in progression** | Number of molecular determinations performed | (*) |
| **171** |  | **Sample analysis technique** | Techniques available for analysis | (4)(*) |
| **172** | **172.1** | **Molecular marker(s) studied** *All biomarkers that are studied must be indicated | List of specific biomarkers for CP | (4)(*) |
|  | **172.2** |  | Others | (1) |
|  | **172.3** |  | None | (1) |
| **173** | **173.1** | **Molecular marker(s) present in the sample** *All biomarkers present must be indicated | List of specific biomarkers for CP | (1) |
|  | **173.2** |  | Others | (1) |
|  | **173.3** |  | None | (1) |
| **174** |  | **Date of molecular determination** | DDMMYYYY | (1)(*) |
| **175** |  | **Molecular report issue date** | DDMMYYYY | (*) |
| **176** |  | **Treatment start date** | DDMMYYYY | (*) |
| **177** | **177.1** | **Type of treatment** | Surgery: procedure | (*) |
|  | **177.2** |  | Chemotherapy: drug/s | (*) |
|  | **177.3** |  | Immunotherapy: drug/s | (*) |
|  | **177.4** |  | Radiotherapy: procedure | (*) |
|  | **177.5** |  | Percutaneous techniques: procedure | (*) |
|  | **177.6** |  | Others: description | (*) |
| **178** |  | **Treatment end date** | DDMMYYYY | (*) |
| The sources marked with (*) are those provided by the members of the Working Groups. | | | | |

**Exitus**

| ***Table 14 . Variables related to exitus*** | | | | |
| --- | --- | --- | --- | --- |
| **Code** | | **Variables** | **Values** | **Reference** |
| **179** |  | **Last contact date** | DDMMYYYY | (1) |
| **180** |  | **Date of death** | DDMMYYYY | (1)(*) |
| **181** | **181.1** | **Cause of death** | Cancer | (1) |
|  | **181.2** |  | Not cancer | (1) |
| **182** |  | **Cause of death code** | According to ICD-9, ICD-10, ICD-11 | (1) |
| **183** | **183.1** | **ICD edition on cause of death** | 09 = ICD-9 | (1) |
|  | **183.2** |  | 10= ICD-10 | (1) |
|  | **183.3** |  | 11 = ICD-11 | (*) |
| The sources marked with (*) are those provided by the members of the Working Groups. | | | | |

**Bibliography**

1. Red Española de Registros de Cáncer (REDECAN), Sociedad Española de Oncología Médica (SEOM). Propuesta de variables de interés para su registro en registros del cáncer de base poblacional y para estudios de alta resolución. 2023 [citado 21 de junio de 2023]; Disponible en: https://seom.org/images/ESTUDIO_REGISTRO_DE_CANCER_BASE_POBLACIONAL.pdf

2. Aller MB, Vázquez ML, Vargas I, Vargas H, Coderch J, Calero S, et al. Indicadores de coordinación asistencial entre niveles de atención. Documento de trabajo. Consorci de Salut i Social de Catalunya [Internet]. 2012. Disponible en: http://www.consorci.org/coneixement/cataleg-de-publicacions/80/indicadores-de-

3. Gobierno de España. Real Decreto 572/2023, de 4 de julio, por el que se modifica el Real Decreto 1093/2010, de 3 de septiembre, por el que se aprueba el conjunto mínimo de datos de los informes clínicos en el Sistema Nacional de Salud. «BOE» núm. 159, de 5 de julio de 2023. 2023 [citado 21 de junio de 2023]; Disponible en: https://www.boe.es/diario_boe/txt.php?id=BOE-A-2023-15551

4. Gobierno de España. Real Decreto 1093/2010, de 3 de septiembre, por el que se aprueba el conjunto mínimo de datos de los informes clínicos en el Sistema Nacional de Salud. «BOE» núm. 225, de 16 de septiembre de 2010. 2010 [citado 21 de junio de 2023]; Disponible en: https://www.boe.es/buscar/doc.php?id=BOE-A-2010-14199

5. Isla D, Lozano MD, Paz-Ares L, Salas C, de Castro J, Conde E, et al. New update to the guidelines on testing predictive biomarkers in non-small-cell lung cancer: a National Consensus of the Spanish Society of Pathology and the Spanish Society of Medical Oncology. Clinical and Translational Oncology. 26 de diciembre de 2022;25(5):1252-67.
